# The Modified Spare Piriformis and Internus, Repair Externus Approach for Hip Arthroplasty

**DOI:** 10.7759/cureus.34999

**Published:** 2023-02-14

**Authors:** Veenesh Selvaratnam, Shalina Gunainthran, Izyan I Akmal, Ahmad Fauzey Kassim

**Affiliations:** 1 Joint Reconstruction Unit (JRU) National Orthopaedic Centre of Excellence for Research and Learning (NOCERAL) Department of Orthopaedic Surgery, Faculty of Medicine, Universiti Malaya, Kuala Lumpur, MYS; 2 Arthroplasty Unit, Sultanah Bahiyah Hospital, Alor Setar, MYS; 3 Arthroplasty Unit, Tengku Ampuan Afzan Hospital, Kuantan, MYS

**Keywords:** thr, posterior approach, hip arthroplasty, nof fracture, spaire, modified spaire

## Abstract

We present the case of an 80-year-old Malaysian gentleman who sustained a displaced intracapsular neck of femur (NOF) fracture and underwent a modified SPAIRE (Sparing Piriformis and Internus, Repair Externus) approach for total hip replacement (THR). The standard approach used in our hospital for THR in NOF fractures is the modified Hardinge approach to the hip. We used this modified SPAIRE approach as this patient lives in a ‘Mahjong’ center and always sits cross-legged on the floor. Therefore, he is at increased risk of an anterior dislocation. This approach is a modification of the standard SPAIRE approach popularized by the Exeter Hip Unit, United Kingdom. In this report, we describe the modification of the SPAIRE approach that has not been described before and the outcome for this patient.

## Introduction

The incidence of intracapsular neck of femur (NOF) fractures continues to rise worldwide [1]. Total hip replacement (THR) is recommended for patients who are independently mobile, have no cognitive impairment, and are fit for anaesthesia. The risk of THR dislocation is higher in patients with NOF fractures compared to patients who have a THR for primary osteoarthritis (OA) [2]. This is due to a more lax capsule in patients with NOF fractures.

Dislocation post-THR is multifactorial, and the incidence varies between 3% and 5% in NOF fractures. The risk factors for dislocation post-THR are implant malposition, spinal pathologies, prior spinal fusion, prior hip surgery, increased age, neuromuscular disease, and high alcohol intake [3]. There are conflicting reports with regard to the association between dislocation rate and the approach used for THR [4]. The currently preferred approach to the hip for a THR in the NOF fracture cohort in our unit is the modified Hardinge anterolateral hip approach.

In 2016, the Exeter Hip Unit in the United Kingdom popularized the SPAIRE (Spare Piriformis and Internus, Repair Externus) approach for hip arthroplasty procedures [5]. This is a tendon-sparing posterior approach to the hip that only involves the division of the obturator externus tendon and part of the quadratus femoris muscle from their femoral insertions. The tendon insertions of the piriformis, superior gemellus, obturator internus, and inferior gemellus are spared. In this article, we describe a modification of the SPAIRE approach for hip arthroplasty surgery. This modified SPAIRE approach is much easier to use and gives better exposure to the acetabulum than the original SPAIRE approach. It also prevents injury to the inferior gemellus muscle bulk.

## Case presentation

**Figure 1:**
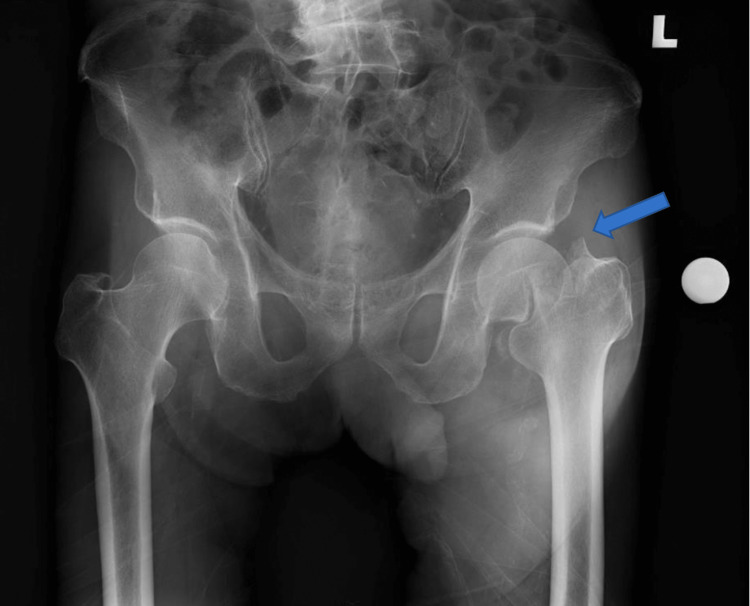
AP radiograph showing a left neck of femur fracture (arrow).

**Figure 2:**
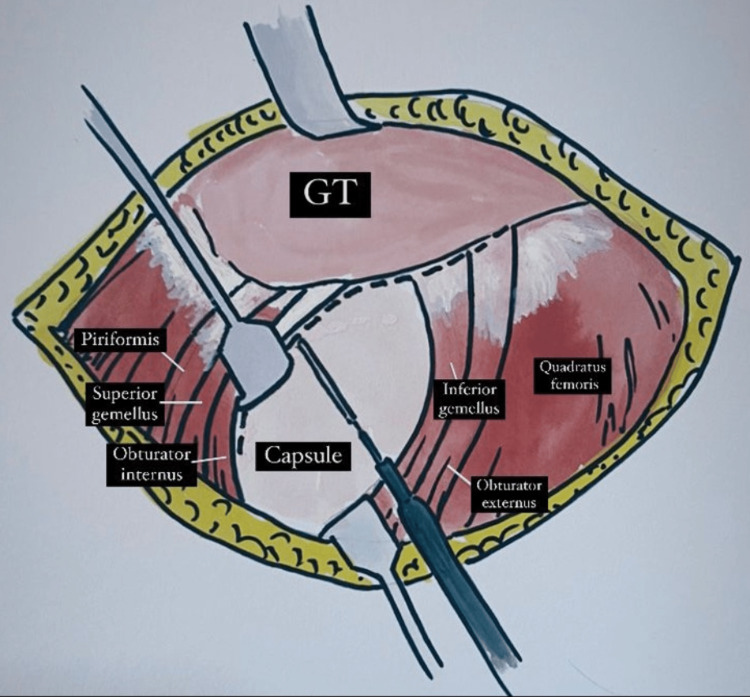
This image shows a Langenbeck retractor retracting and protecting the obturator internus, superior gemellus, and piriformis. It also shows the line of incision along the capsule, inferior gemellus and obturator externus using a diathermy to expose the hip joint in this modified SPAIRE approach.

**Figure 3:**
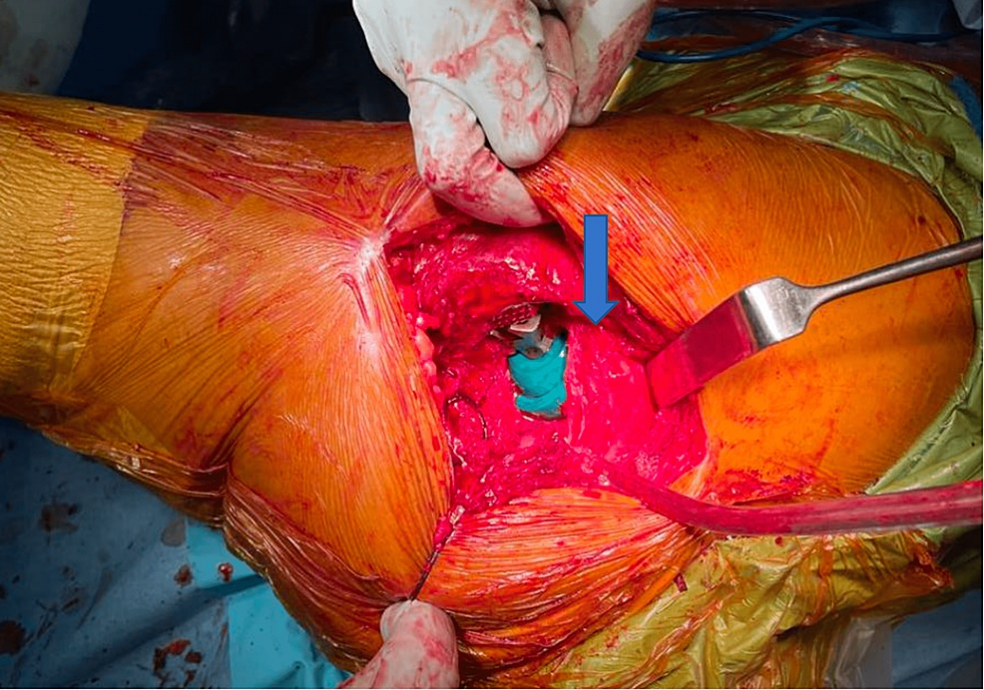
Intra-operative clinical picture showing the modified SPAIRE approach with preservation of the piriformis, superior gemellus, and obturator internus (arrow).

**Figure 4:**
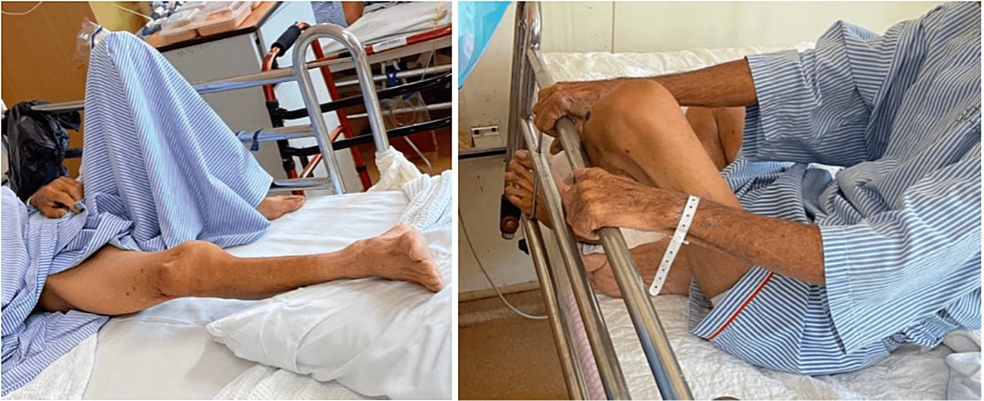
Patient was flexing his left hip up to 90 degrees and sitting cross-legged day 1 post op.

**Figure 5:**
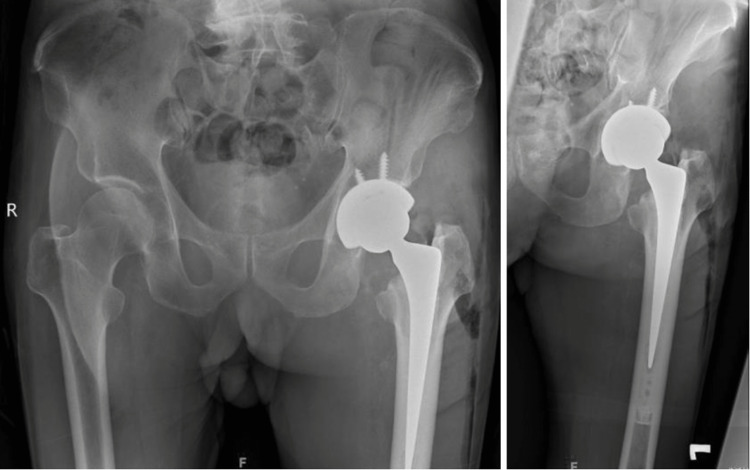
Post-operative AP pelvis and AP left hip radiographs showing a satisfactory left total hip replacement using the modified SPAIRE approach.

This was an 80-year-old gentleman who is normally fit and well. He fell off his bicycle after he was chased by a pack of dogs. He sustained an isolated displaced left intracapsular NOF fracture (Figure 1). He was independently mobile prior to his injury. He lives in a ‘Mahjong’ center and always sits cross-legged on the floor, which puts him at a higher risk of anterior dislocation. While he was an inpatient awaiting surgery, we noticed that he was not a very reliable patient and that he was not compliant with nursing and medical care. He kept his right leg flexed and externally rotated at all times. He said he normally keeps his left leg in this position too, as he always sits cross-legged. He was not able to keep still, and he did not follow instructions. From his behavior, we knew that he would not follow post-operative hip precautions. He was at a higher risk of an anterior dislocation rather than a posterior dislocation due to his preferred sitting position. Therefore, a decision was made to perform a modified SPAIRE approach to the hip. The modified SPAIRE approach used was similar to that described by Hanly et al. [5] except we released the inferior gamellus, obturator externus, and part of the quadratus femoris (Figure 2) and used conventional retractors as we do not have specialised designated SPAIRE® hip instruments. The patient was positioned in the right lateral position (Figure 3). A standard incision for a posterior approach was used, centered at the posterolateral tip of the greater trochanter. The trochanteric bursa was swept posteriorly to expose the short external rotators. The sciatic nerve was identified and protected. The internal space between the obturator internus and inferior gemellus was identified. The plane between the obturator internus, superior gemellus, piriformis, and capsule was defined using Mayo scissors. A 90-degree Langenbeck retractor was placed in this plane to retract the obturator internus, superior gemellus, and piriformis. The obturator externus and inferior gemellus were detached from the greater trochanter together with an L-shaped posterior capsulotomy with the proximal oblique limb at 2 o’clock (10 o’clock for a right hip) using diathermy. Abduction of the hip past the neutral position with the knee lifted helps with this step as it relaxes the piriformis, superior gemellus, and obturator internus. The detached capsule, obturator externus, and inferior gemellus were held with braided nonabsorbable stay sutures, which were used for transosseous posterior repair at the end of the procedure. We used a standard hybrid THR (uncemented acetabular component and a cemented taper slip stem) for this patient. The stability assessment was checked, as is normally done for a routine posterior approach. The remaining short external rotators prevented posterior dislocation during trialing, and a dislocation hook had to be used to dislocate the hip. The surgery was uneventful. The patient mobilized with crutches the following day and was discharged three days post-operatively. As expected, this patient did not follow any post-operative hip precautions and was flexing his hip more than 90 degrees immediately post-surgery and was sitting crossed-legged (Figure 4). His post-operative radiographs were satisfactory (Figure 5). His Oxford Hip Score at three months post-operation was 44, and he was happy with his hip. He defaulted on all further follow-ups, and he is now two years post-surgery.

## Discussion

The inferior gemellus is a muscular structure rather than tendinous like the obturator internus. It therefore almost always tears due to retraction to expose the hip joint when performing the SPAIRE approach. Therefore, we propose the modified SPAIRE technique. In the modified SPAIRE technique, the inferior gemellus is released together with the obturator externus and part of the quadratus femoris at the femoral insertion when performing this approach. By releasing the inferior gemellus, it provides better exposure and also preserves the inferior gemellus from getting damaged due to retraction. During the closure, the inferior gemellus, along with the obturator externus and posterior capsule, can be repaired with strong non-absorbable sutures through an enhanced trans-osseous technique onto the posterior aspect of the greater trochanter.

The original SPAIRE approach described is done with the utilization of the SPAIRE® Hip Instruments by Platts & Nisbett (Sheffield, UK). In most centres around the world, this set is not available; therefore, the SPAIRE approach can only be done with conventional hip retractors. We use a 90-degree Langenbeck retractor to retract the remaining preserved short external rotators. The short external rotators and the hip capsule are laxer in NOF fracture patients compared to primary hip OA patients. Therefore, it is almost impossible to use the SPAIRE approach when performing a primary THR in OA patients without the designated SPAIRE® Hip instruments. 

The first author in this paper, who was fellowship trained in the Exeter Hip Unit, routinely performs the modified SPAIRE approach for hip hemiarthroplasty cases and selected THR for NOF fracture patients without the designated SPAIRE® Hip instruments. This approach can be challenging with overweight patients and inexperienced assistants. For someone starting to use this approach, it is best done in a NOF fracture patient needing a bipolar hip hemiarthroplasty.

The SPAIRE approach has been shown to be superior to the standard lateral approach in patients who underwent hip hemiarthroplasty after a NOF fracture in terms of patients returning to their pre-injury level of mobility [6]. In the Exeter Hip Unit, patients who have their THR using the SPAIRE approach do not need to follow any hip precautions. We do not advocate any hip precautions in post-op patients who have undergone THR or hip hemiarthroplasty using the modified SPAIRE approach. This is because of the tremendous stability provided by the preserved short external rotators, especially the obturator internus.

The piriformis sparing technique in THR with a posterior approach has shown to be beneficial in terms of stability [7], but the modified SPAIRE approach is a different beast in terms of stability obtained due to the preserved piriformis, superior gemellus, and obturator internus. A dislocation hook is needed to dislocate the hip during trailing, and this stability is not achieved with just the piriformis sparing technique. We routinely use the piriformis sparing technique for THR in primary hip OA cases.

## Conclusions

Generally, the success of a THR is dependent on its ability to restore hip biomechanics and function and alleviate pain associated with hip pathology. There are many documented and described approaches to the hip. The approach utilized should allow adequate exposure for the surgeon to reproducibly and reliably place the THR implants to restore hip anatomy and function while decreasing the risk of surgical complications. This modification has not been described elsewhere. This modification is more 'user friendly' and provides better exposure to the hip joint, especially during acetabular preparation and implantation when using the SPAIRE approach. It also prevents damage to the inferior gemellus due to retraction while maintaining the tremendous stability obtained from using the SPAIRE approach.
